# Targeting flavin-containing enzymes eliminates cancer stem cells (CSCs), by inhibiting mitochondrial respiration: Vitamin B2 (Riboflavin) in cancer therapy

**DOI:** 10.18632/aging.101351

**Published:** 2017-12-16

**Authors:** Bela Ozsvari, Gloria Bonuccelli, Rosa Sanchez-Alvarez, Richard Foster, Federica Sotgia, Michael P. Lisanti

**Affiliations:** ^1^ Translational Medicine, School of Environment and Life Sciences, Biomedical Research Centre (BRC), University of Salford, Greater Manchester, UK; ^2^ The Paterson Institute, University of Manchester, Withington, UK; ^3^ School of Molecular & Cellular Biology and Astbury Centre for Structural Molecular Biology, Faculty of Biological Sciences, University of Leeds, West Yorkshire, UK; ^4^ School of Chemistry, Faculty of Mathematics and Physical Sciences, University of Leeds, West Yorkshire, UK

**Keywords:** Vitamin B2 (Riboflavin), DPI (Diphenyleneiodonium chloride), mitochondria, Mitoflavoscin, flavin enzymes, OXPHOS, cancer stem-like cells (CSCs)

## Abstract

Here, we performed high-throughput drug-screening to identify new non-toxic mitochondrial inhibitors. This screening platform was specifically designed to detect compounds that selectively deplete cellular ATP levels, but have little or no toxic side effects on cell viability. Using this approach, we identified DPI (Diphenyleneiodonium chloride) as a new potential therapeutic agent. Mechanistically, DPI potently blocks mitochondrial respiration by inhibiting flavin-containing enzymes (FMN and FAD-dependent), which form part of Complex I and II. Interestingly, DPI induced a chemo-quiescence phenotype that potently inhibited the propagation of CSCs, with an IC-50 of 3.2 nano-molar. Virtually identical results were obtained using CSC markers, such as CD44 and CD24. We further validated the effects of DPI on cellular metabolism. At 10 nM, DPI inhibited oxidative mitochondrial metabolism (OXPHOS), reducing mitochondrial driven ATP production by >90%. This resulted in a purely glycolytic phenotype, with elevated L-lactate production. We show that this metabolic inflexibility could be rapidly-induced, after only 1 hour of DPI treatment. Remarkably, the mitochondrial inhibitory effects of DPI were reversible, and DPI did not induce ROS production. Cells maintained in DPI for 1 month showed little or no mitochondrial activity, but remained viable. Thus, it appears that DPI behaves as a new type of mitochondrial inhibitor, which maintains cells in a state of metabolic-quiescence or “suspended animation”. In conclusion, DPI treatment can be used to acutely confer a mitochondrial-deficient phenotype, which we show effectively depletes CSCs from the heterogeneous cancer cell population. These findings have significant therapeutic implications for potently targeting CSCs, while minimizing toxic side effects. We also discuss the possible implications of DPI for the aging process. Interestingly, previous studies in *C. elegans* have shown that DPI prevents the accumulation of lipofuscin (an aging-associated hallmark), during the response to oxidative stress. Our current results are consistent with data showing that flavins (FAD, FMN and/or Riboflavin) are auto-fluorescent markers of i) increased mitochondrial “power” (OXPHOS) and ii) elevated CSC activity. Finally, we believe that DPI is one of the most potent and highly selective CSC inhibitors discovered to date. Therefore, our current findings suggest a new impetus to create novel analogues of i) DPI (Diphenyleneiodonium chloride) and ii) DPI-related compounds (Diphenyliodonium chloride), using medicinal chemistry, to optimize this very promising and potent anti-CSC activity. We propose to call these new molecules “Mitoflavoscins”. For example, DPI is ∼30 times more potent than Palbociclib (IC-50 = 100 nM), which is an FDA-approved CDK4/6 inhibitor, that broadly targets proliferation in any cell type, including CSCs.

## INTRODUCTION

Cancer stem-like cells (CSCs) are thought to be one of the main drivers of poor clinical outcome, in a wide variety of tumor types and especially in advanced disease states. This is because CSCs are responsible for initiating the process of treatment failure, ultimately resulting in tumor recurrence and distant metastasis [1–4]. Unfortunately, current conventional therapies were not designed to target CSCs; so, new drug development in this clinical area is warranted and is a high translational priority [5].

Recently, we proposed that CSCs have an increased mitochondrial mass and are functionally dependent on mitochondrial biogenesis and OXPHOS for their successful propagation, in an anchorage-independent fashion [6–8]. In support of this mechanistic hypothesis, we showed that FDA-approved antibiotics that behave as inhibitors of mitochondrial biogenesis (Azithro-mycin, Doxycycline and Tigecycline) or OXPHOS (Pyrvinium pamoate, Atovaquone and Bedaquiline), all effectively restrict the propagation of CSCs [9–13]. Other FDA-approved drugs (or dietary supplements) with anti-OXPHOS activity, also targeted CSCs (Berberine, Irinotecan, Sorafenib and Niclosamide) [14]. Similarly, the warfarin-like drug Dicoumarol, was sufficient to overcome tamoxifen-resistance in ER(+) cancer cells, by targeting the protective effects of NQO1 on cancer cell mitochondria [15].

Because of these promising findings, here we decided to intensify our search for new metabolic inhibitors, by screening a library of FDA-approved drugs and other related test compounds, with known targets and established mechanisms of action. More specifically, we restricted our search to compounds that significantly reduced ATP production, but did not induce cell death, to avoid drugs with acute toxic side-effects.

This focused approach allowed us to identify DPI as a fast-acting, reversible and “non-toxic” mitochondrial inhibitor, which preferentially targets the propagation of CSCs. Remarkably, DPI induces a “chemo-quiescence” phenotype, but without resulting in massive cell death. DPI prevented the propagation of CSCs, with an IC-50 of ∼3 nM, without reducing the viability of the total “bulk” cancer cell population or normal fibroblasts.

Furthermore, DPI selectively reduced the CD44+/CD24- CSC population, with an IC-50 of ∼10 nM.

## RESULTS

### Screening for novel “non-toxic” mitochondrial inhibitors: identification of DPI as a top hit compound

**Figure 1:**
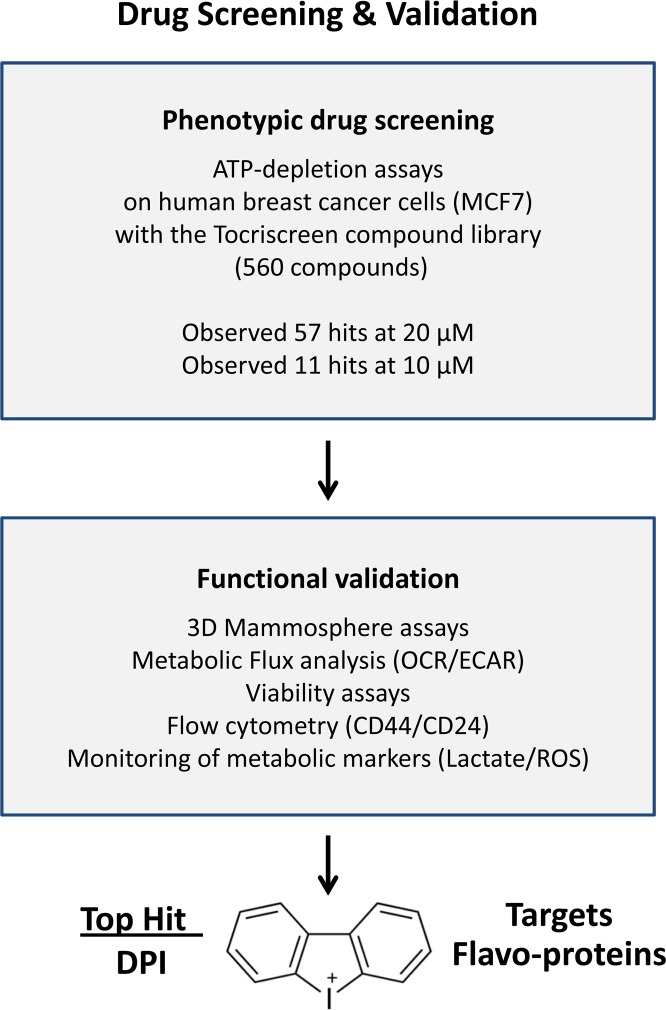
Diagram illustrating the main steps of our drug discovery work-flow **(i) Phenotypic drug screening.** A sub-set of the Tocriscreen compound library was subjected to phenotypic drug screening, at a concentration of 20 μM. The screen was set up to specifically identify compounds which can functionally induce ATP-depletion, without inducing cell death. Subsequently, positive hits were re-screened at a lower concentration (10 μM). **(ii) Functional validation.** Hit compounds were then further validated using mammosphere assays (for assessing potential anti-cancer stem cell activity). Metabolic flux analysis (to determine specific effects on oxygen consumption), flow cytometry and viability assays were also carried out. **(iii) Top hit compound.** The structure of DPI (Diphenyleneiodonium chloride), the top hit compound, is shown. Importantly, DPI is known to functionally target flavin-containing enzymes, especially within mitochondrial complex I (NDUFV1/2/3) and II (SDHA), as well as the TCA cycle. DPI chemically reacts with and inactivates FMN (flavin mononucleotide).

Recently, we developed a new approach for the therapeutic targeting of CSCs, focused specifically on mitochondrial metabolism. A schematic diagram summarizing this metabolic approach to drug discovery and functional validation is illustrated in Figure 1.

Briefly, the screening assay was designed to identify compounds, which can functionally induce ATP-depletion, but without inducing cell death, to avoid toxic side-effects. Initially, a sub-set of the Tocriscreen Compound library (>500 chemical entities) was subjected to phenotypic drug screening, at a concentration of 20 μM. Subsequently, positive hits were re-screened at a lower concentration (10 μM).

As DPI (Diphenyleneiodonium chloride) was identified as our top hit compound, we subjected it to further functional validation, including: i) mammosphere assays (for assessing potential anti-cancer stem cell activity); ii) metabolic flux analysis (to determine specific effects on oxygen consumption), as well as iii) flow cytometry (to monitor the expression of well-established CSC markers).

**Figure 2:**
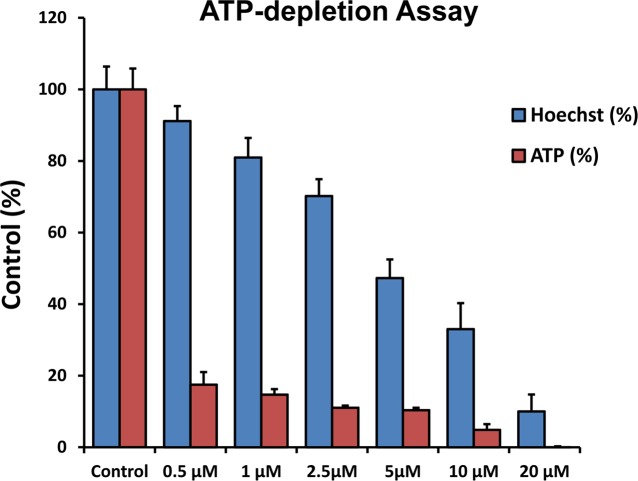
DPI selectively depletes ATP without inducing massive cell death MCF7 cells were treated with DPI for 72 hours and were first subjected to fluorescent Hoechst staining (DNA content) and then to the luminescent measurement of the ATP content in the same wells, using CellTiter-Glo as a probe. Note that at 72 hours, 500 nM DPI selectively depletes ATP levels by >80%, but does not significantly induce cell death, as the number of cells attached to the plate remains the unchanged (as detected by DNA content).

Figure 2 demonstrates that DPI selectively depletes ATP levels, without inducing massive cell death. Briefly, human breast cancer cells (MCF7) were treated with DPI for 72 hours. Then, they were subjected to fluorescent Hoechst DNA-staining (to normalize for cell number); by employing CellTiter-Glo as a probe, we were able to use luminescence to measure ATP content in the same wells. Importantly, at 72 hours of treatment, 500 nM DPI dramatically reduced ATP levels, but did not significantly induce any cell death, as the number of cells attached to the plate remained unchanged (as detected by DNA content).

**Figure 3:**
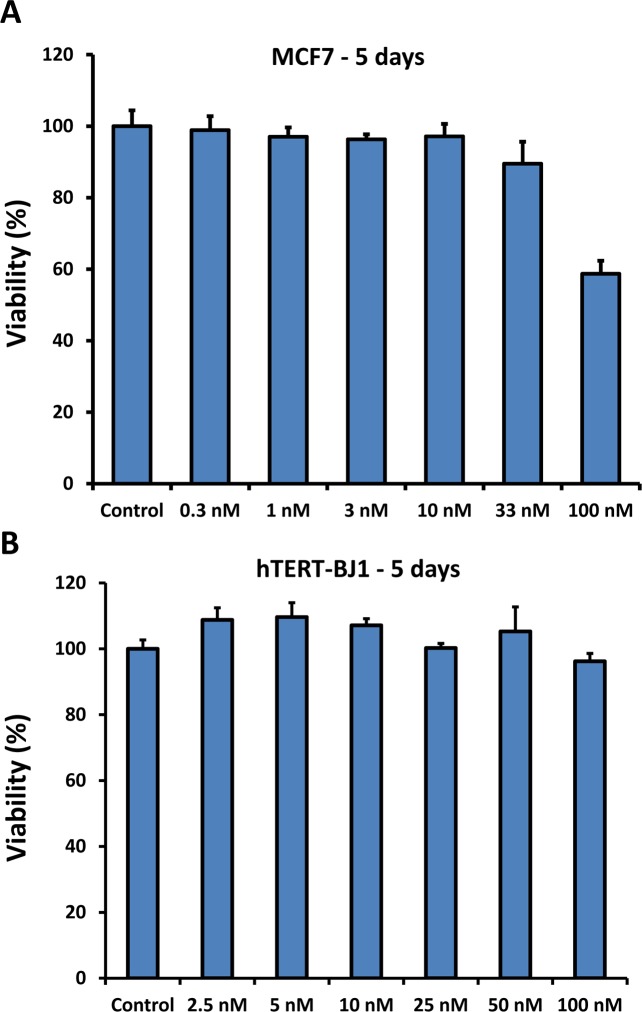
DPI does not significantly affect cell viability, even after 5 days of treatment Cell viability was determined by employing the Sulphorhodamine B (SRB) assay, to measure total protein content. Note that after 5 days of incubation, DPI shows little or no toxicity in MCF7 cells, at a concentration as high as 33 nM. However, some toxicity was observed at 100 nM. Similarly, normal fibroblasts (hTERT-BJ1) showed little or no toxic effects at up to 100 nM, after 5 days of incubation.

In parallel, cell viability assays were carried out to evaluate its potentially toxicity. Figure 3 shows that DPI does not significantly affect cell viability, even after 5 days of treatment. Cell viability was assessed by employing the SRB-assay, which measures protein content. After 5 days of treatment, DPI showed little or no toxicity in MCF7 cells, at a concentration as high as 33 nM. Virtually identical results were also obtained with normal fibroblasts (hTERT-BJ1), which showed little or no toxic effects, at up to 100 nM, even after 5 days of incubation.

### Validation of DPI as a potent mitochondrial inhibitor

**Figure 4:**
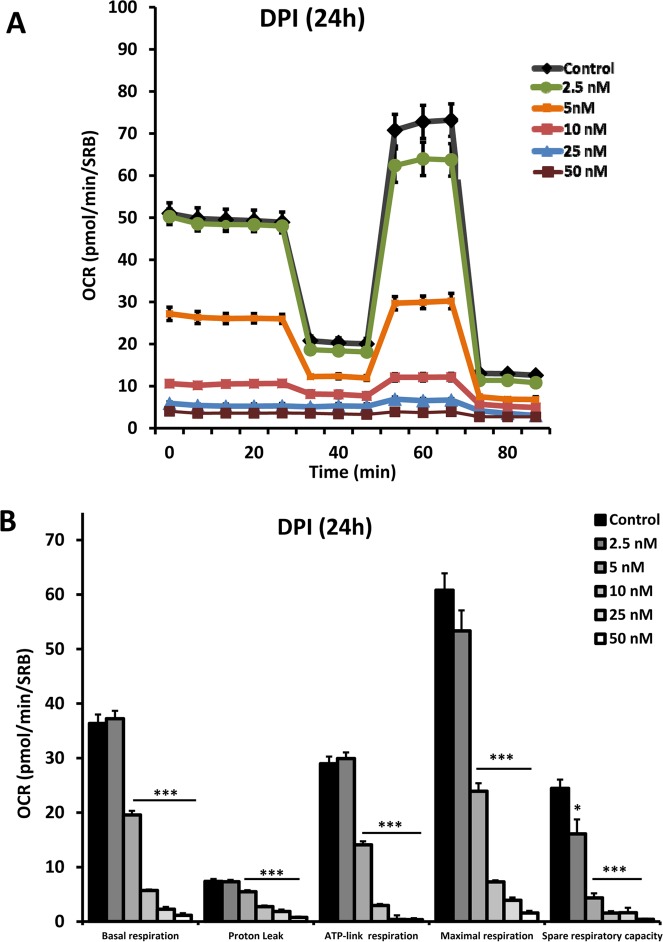
DPI potently inhibits mitochondrial respiration After 24 hours of treatment with DPI (2.5 to 50 nM), MCF7 cells were subjected to metabolic flux analysis with the Seahorse XFe96, which measures the OCR (the oxygen consumption rate). Note that at concentration of 2.5 nM, little or no effect was observed. However, at 5 nM, basal respiration was reduced by ∼ 50%. ^***^ p<0.001.

To functionally validate that DPI behaves as a strong mitochondrial inhibitor, we subjected DPI-treated breast cancer cells to a “mitochondrial stress test”. Figure 4 illustrates that DPI potently inhibited mitochondrial respiration. First, MCF7 cells were exposed to 24 hours of treatment with DPI, at a concentration of 2.5 to 50 nM. Then, the treated cells were analyzed with the Seahorse XFe96, which measures the OCR (the oxygen consumption rate). Importantly, at a concentration of 2.5 nM, DPI had little or no effect. However, at 5 nM, basal respiration was reduced by ∼ 50%. Finally, at 10 nM, the basal respiration rate was decreased by ∼85%, resulting in a >90% reduction in ATP production.

### Induction of glycolysis and L-lactate production by DPI

**Figure 5:**
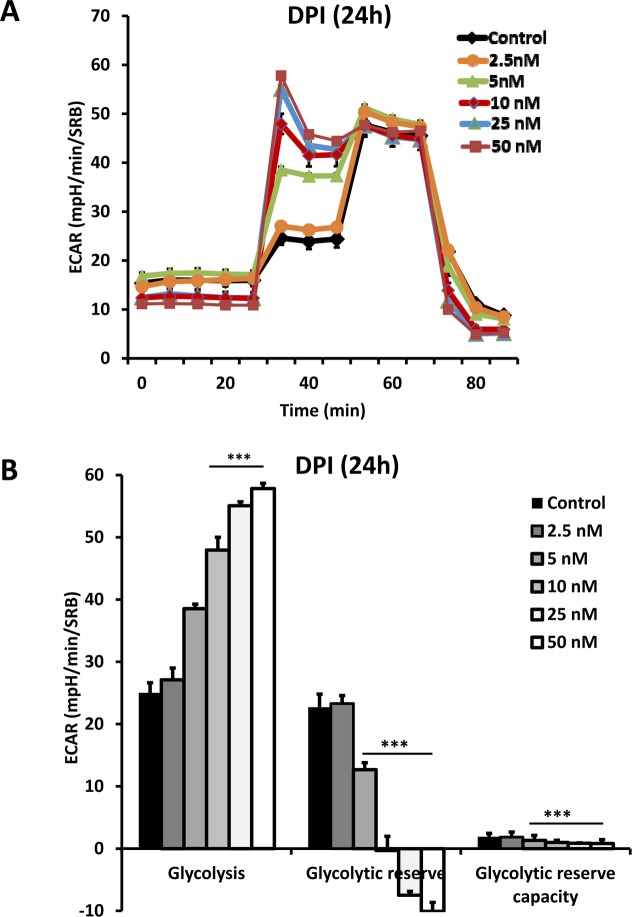
DPI induces a reactive glycolytic response After 24 hours of treatment with DPI (2.5 to 50 nM), MCF7 cells were subjected to metabolic flux analysis with the Seahorse XFe96, which also measures ECAR (the extracellular acidification rate), a surrogate marker for L-lactate production. Note that at a concentration of 2.5 nM, little or no effect was observed. However, at 10 nM, glycolysis was increased by 2-fold. ^***^ p<0.001.

To determine if the anti-mitochondrial effects of DPI induce a reactive glycolytic response, we next subjected DPI-treated breast cancer cells to a “glycolytic stress test”. Figure 5 highlights that DPI potently induced a glycolytic phenotype. MCF7 cells were subjected to metabolic flux analysis with the Seahorse XFe96, which also measures ECAR (the extracellular acidification rate), as a surrogate marker for L-lactate production. After 24 hours of treatment with DPI (2.5 nM), little or no effect was observed. However, at 10 nM DPI, glycolysis was increased by ∼2-fold.

To confirm that the increased ECAR we observed corresponded to L-lactate production, L-lactate levels were measured directly using the ISCUS-flex microdialysis analyser. After treatment with DPI (5 or 50 nM) for 1, 3 or 5 days, the cell culture media from MCF7 cells was used to measure L-lactate content.

**Figure 6:**
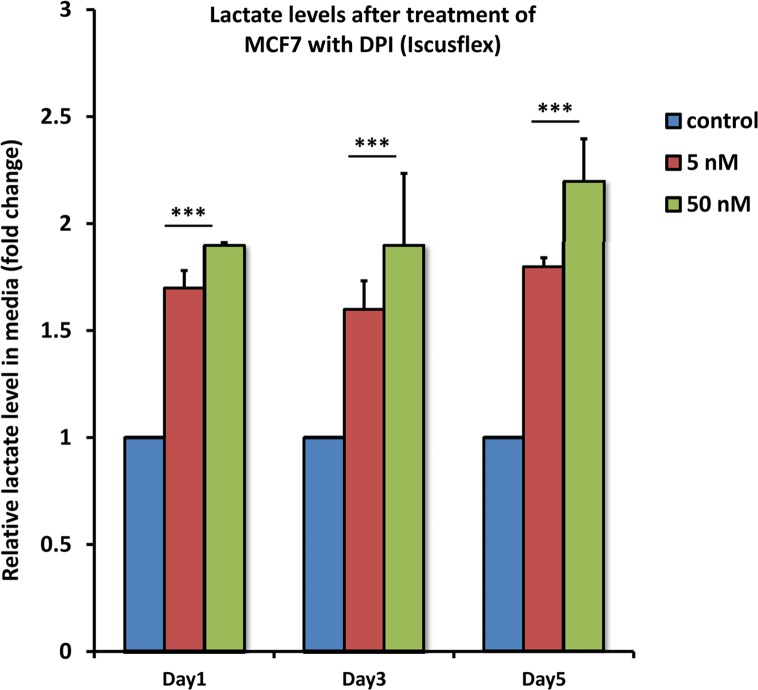
DPI drives the production of L-lactate After treatment with DPI (5 or 50 nM) for 1, 3 or 5 days, the cell culture media from MCF7 cells was subjected to analysis using the ISCUS-flex microdialysis analyser, to directly measure L-lactate content. Note that DPI induces significant L-lactate production, nearly doubling the amount of lactate as early as 1 day of treatment, using only 5 nM DPI. ^***^ p<0.001.

Figure 6 shows that DPI induced significant L-lactate production, nearly doubling the amount of lactate produced, consistent with a 2-fold increase in glycolysis.

### DPI treatment effectively inhibits the propagation of CSCs, without increasing ROS production

**Figure 7:**
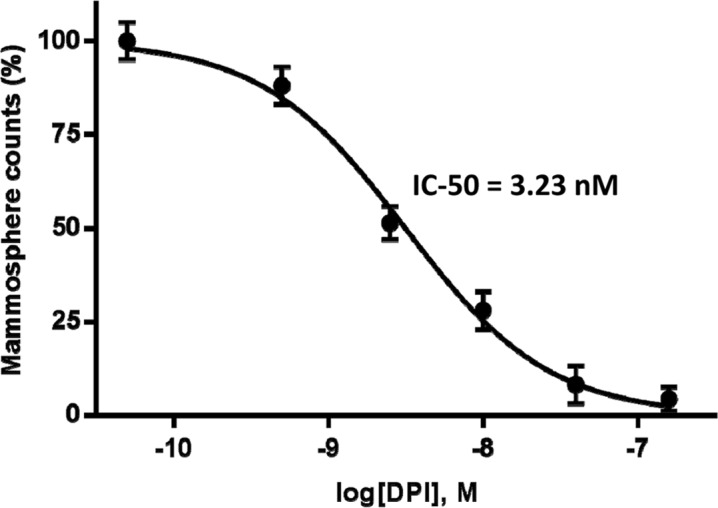
Dose-dependent inhibition of CSC propagation using DPI, as measured using the mammosphere assay MCF7 cells were seeded into low-attachment plates and allowed to form mammospheres (a.k.a., 3D tumor spheres), over a period of 5 days. Note that DPI markedly reduced CSC propagation, with an IC-50 of 3.23 nM. The mammosphere assay was performed over the range of 0.2 nM to 10 μM.

Figure 7 highlights that DPI dose-dependently inhibited CSC propagation, as assessed using the mammosphere (3D tumor sphere) assay. To determine the potential anti-CSC activity of DPI, MCF7 cells were first seeded into low-attachment plates and allowed to form mammospheres over a period of 5 days. The mammosphere assay was performed over the range of 0.2 nM to 10 μM DPI. Remarkably, DPI treatment significantly reduced CSC propagation, in a concentration-dependent manner, with an IC-50 of 3.23 nM.

To further validate our findings, we used a second independent approach to quantitate “stemness” in cancer cells, by employing specific cell surface markers, namely fluorescent antibody probes directed against CD44 and CD24. In this context, the CD44+/CD24- cell population represnts the CSC-enriched fraction.

**Figure 8:**
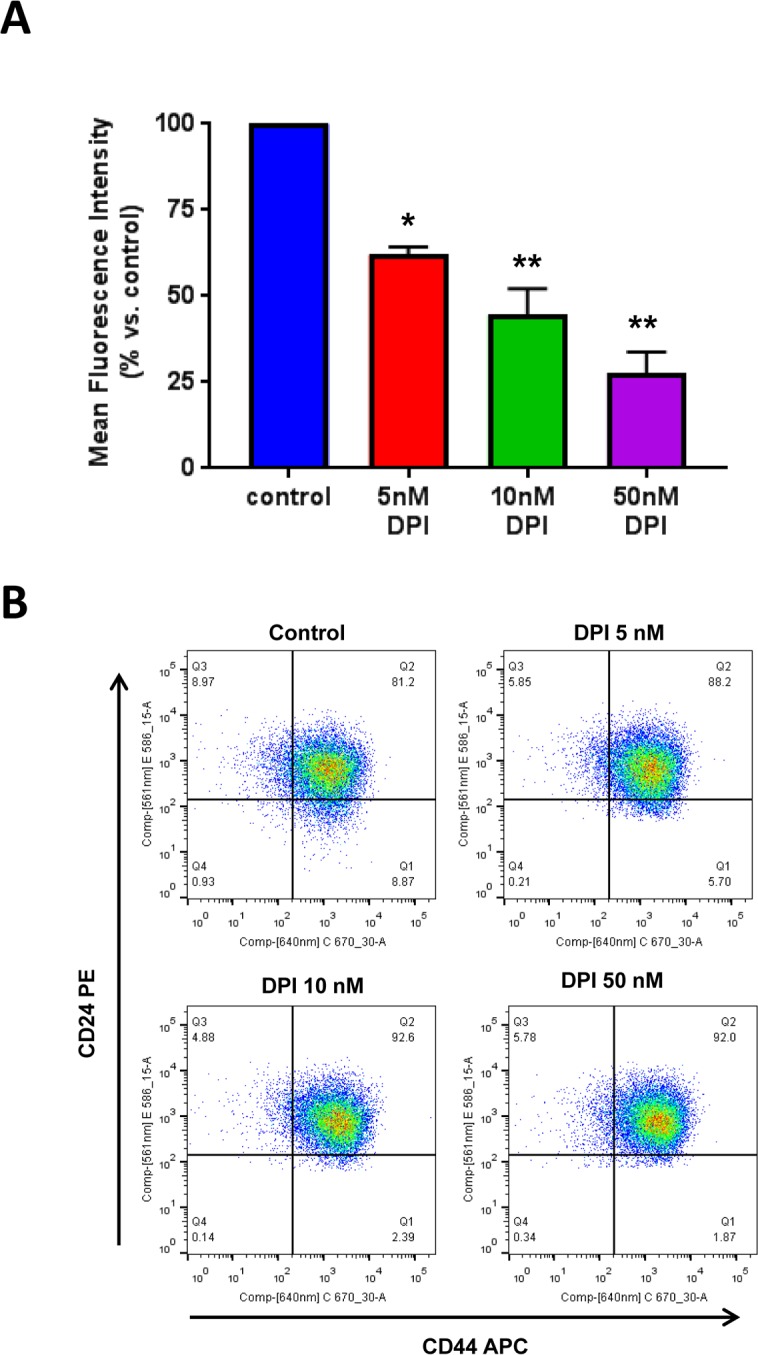
DPI selectively eliminates CSCs from the total cell population MCF7 cells were cultured for 5 days as monolayers, in the presence of DPI (5, 10 and 50 nM). Then, the cells were harvested and subjected to FACS analysis to determine the levels of CSC markers. Panel (**A**) shows that the CD44+/CD24- cell population (which serves as a marker for breast CSCs) is dose-dependently reduced by DPI treatment, with an IC-50 of 10 nM. Panel (**B**) contains dot plots showing the double fluorescent CD44+/CD24- FACS assay. Note that the signal has significantly decreased in the lower right quadrant (Q1), after DPI treatment. ^*^ p<0.05, ^**^ p<0.01.

Figure 8 shows that DPI selectively eliminates these CSCs from the total cell population. Briefly, MCF7 cells were cultured for 5 days as monolayers, in the presence of DPI (5, 10 and 50 nM). Then, the cells were were harvested and subjected to FACS analysis to determine the levels of CSC markers. Interestingly, the CD44+/CD24- cell population was dose-dependently reduced by DPI treatment, with an IC-50 of 10 nM.

One possible mechanism by which DPI inhibits CSC propagation is by simply inducing mitochondrial ROS production. To address this issue, we directly determined the effects of DPI, after 24 hours of treatment, on mitochondrial ROS production, over the range of 5 to 50 nM. Mitochondrial ROS production was monitored by FACS analysis, using MitoSOX as a fluorescent indicator.

**Figure 9:**
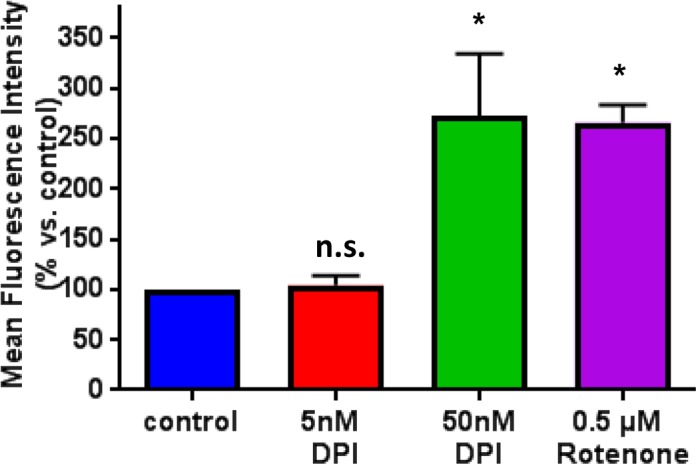
Effect of DPI treatment on mitochondrial ROS production The effects of DPI on mitochondrial ROS production were determined over the range of 5 to 50 nM. Note that at a concentration of 5 nM, DPI failed to induce mitochondrial ROS production. In contrast, 50 nM DPI induced the same amount of mitochondrial ROS as 500 nM Rotenone, which was used as a positive control. Mitochondrial ROS production was monitored by FACS analysis, using MitoSOX as a fluorescent indicator. ^*^ p<0.05.

Figure 9 illustrates that at a concentration of 5 nM, DPI failed to induce any detectable mitochondrial ROS production, relative to control cells, treated with vehicle alone. In contrast, 50 nM DPI induced the same amount mitochondrial ROS as 500 nM Rotenone, which served as a positive control.

Therefore, the same concentration of DPI (5 nM) that inhibited mammosphere formation by >50% failed to increase mitochondrial ROS production. As such, the effects of low-dose DPI on “stemness” in cancer cells cannot be explained simply by ROS production.

**Figure 10:**
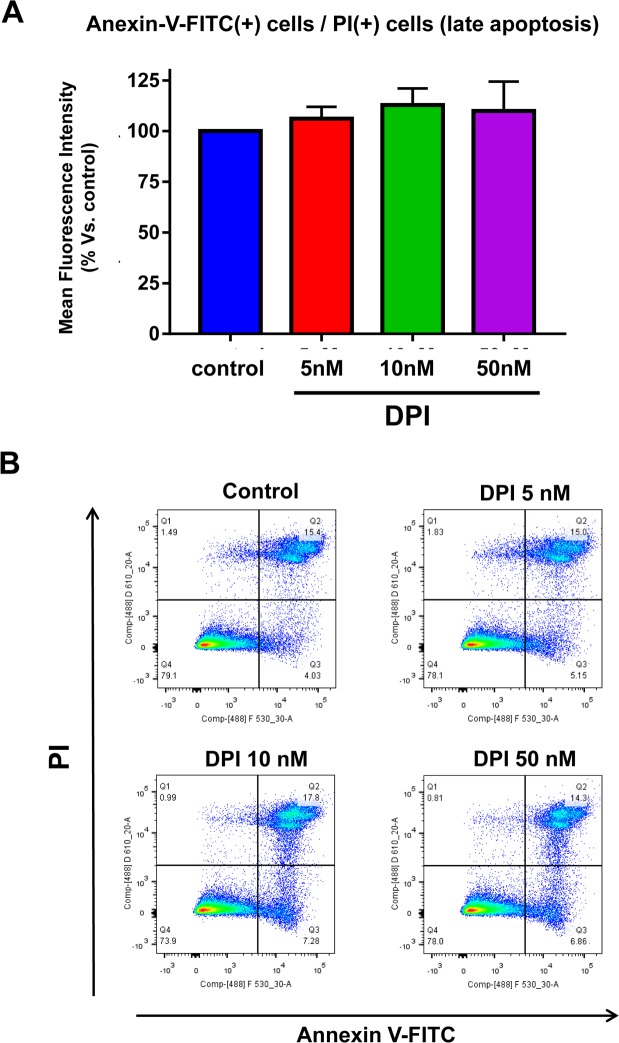
DPI is generally “non-toxic” and does not increase the apoptotic rate in MCF7 cell monolayer cultures Briefly, 300,000 MCF7 cells were plated in 6-well plates in complete media supplemented with 10% HiFBS. On the next day, the cells were treated with DPI (5, 10, or 50 nM) for 24 hours. Vehicle alone (DMSO) for control cells were processed in parallel. At least 30,000 events were recorded by FACS using LSRII. The results presented are the average of three biological replicates analyzed in independent experiments and are expressed as mean fluorescence intensity. (**A**) Bar-graphs are used to summarize the overall results; (**B**) Representative FACS tracings are also shown. Note that DPI fails to significantly increase the apoptotic rate in MCF7 cell monolayers.

In direct support of DPI's high selectivity in the targeting the CSC population, DPI had no effect on programmed cell death (apoptotic rate) in MCF7 cell monolayers, as measured quantitatively by FACS analysis, using a combination of PI and Annexin-V staining (Figure 10).

### DPI is a fast-acting, reversible, “non-toxic” mitochondrial inhibitor

**Figure 11:**
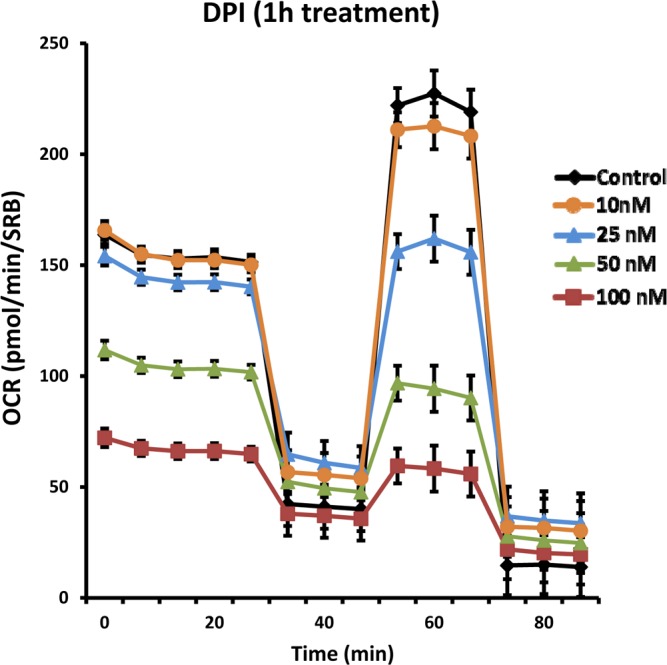
DPI rapidly induces the inhibition of mitochondrial respiration Even with as little as 1 hour of DPI treatment, the mitochondrial oxygen consumption rate (OCR) was progressively reduced, over a concentration range of 10 to 100 nM, as seen using the Seahorse XFe96 Metabolic Flux Analyzer. Note that basal respiration was inhibited with an IC-50 of 50 nM.

Given DPI's high potency, we next assessed its ability to rapidly affect cell metabolism. Figure 11 demonstrates the remarkably fast-action of DPI on mitochondrial respiration. After as little as 1 hour of DPI treatment, the mitochondrial oxygen consumption rate (OCR) was progressively reduced, over a concentration range of 10 to 100 nM. Basal respiration was inhibited with an IC-50 of 50 nM.

**Figure 12:**
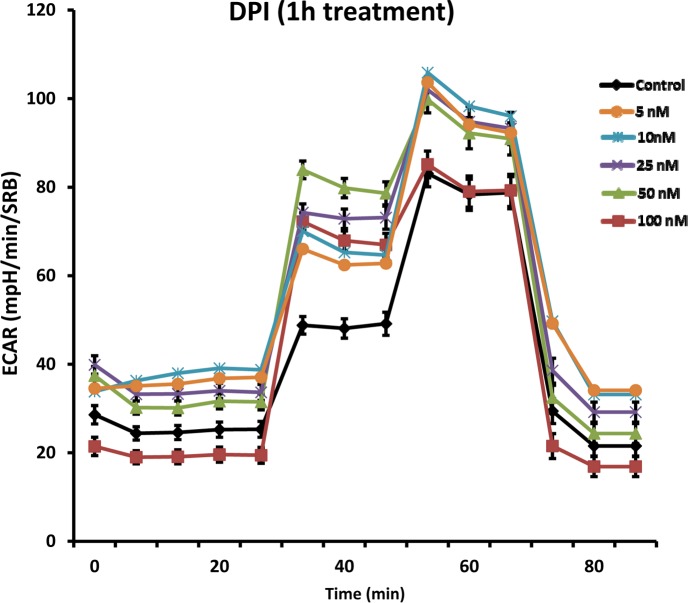
DPI rapidly induces a reactive glycolytic phenotype Even with as little as 1 hour of DPI treatment, glycolysis was progressively increased, over a concentration range of 5 to 100 nM, as seen using the Seahorse XFe96 Metabolic Flux Analyzer. Note that glycolysis was effectively doubled.

Similarly, DPI rapidly induced a reactive glycolytic phenotype. Glycolysis progressively increased, over a concentration range of 5 to 100 nM. Figure 12 shows that glycolysis effectively doubled.

**Figure 13:**
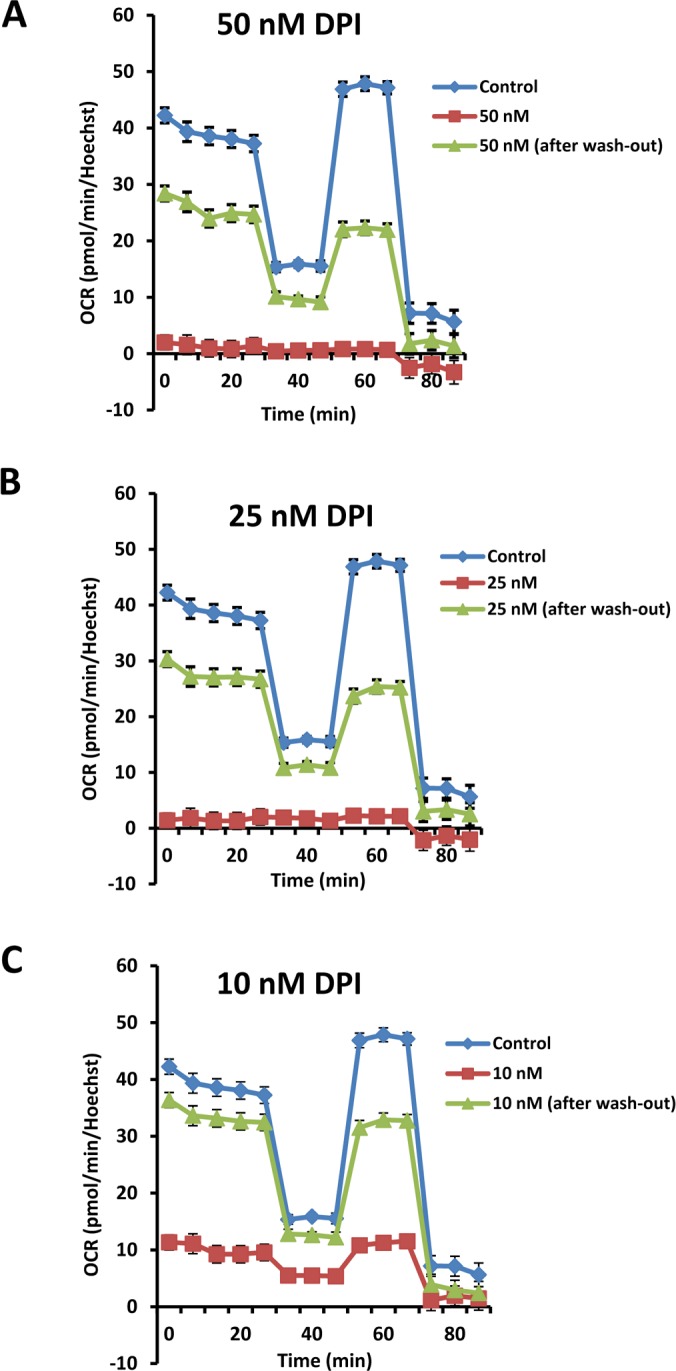
The inhibitory effects of DPI on mitochondrial respiration are reversible To assess the reversibility of DPI's effects after drug removal, MCF7 cells were first subjected to DPI treatment for 24 hours. Then, the DPI was removed by washing with normal media and the cells were cultured for an additional 24 hours to allow them to recover. This cycle of DPI-treatment, “wash-out” and recovery was carried out over a concentration range of 10 to 50 nM DPI. Note that with 10 nM DPI, there was a near complete recovery of basal respiration, after only 24 hours. Higher concentrations (25 and 50 nM) still showed significant recovery, but the recovery was not complete at this time point.

**Figure 14:**
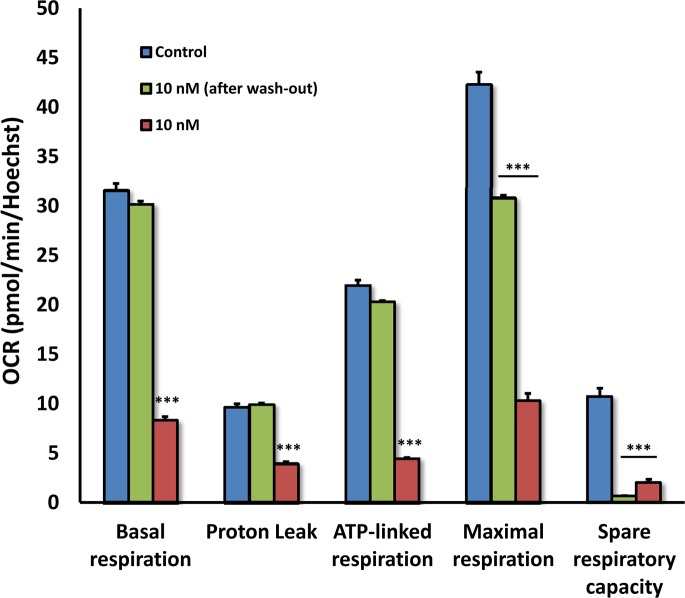
The inhibitory effects of DPI on mitochondrial respiration are reversible: Focus on 10 nM As in Figure 12, except that Panel C is shown instead as a series of bar graphs, to better illustrate and separate the different metabolic parameters.

The effects of DPI also appeared to be highly reversible. To assess the reversibility of DPI's effects, MCF7 cells were first subjected to DPI treatment for 24 hours (Figures 13 and 14). Then, DPI was removed and the cells were cultured for an additional 24 hours, to allow recovery. DPI-treatment, “wash-out” and recovery was performed over a concentration range of 10 to 50 nM DPI. At 10 nM DPI, there was a near complete recovery of basal respiration, approaching 100%, after only 24 hours. Higher concentrations showed significant recovery, but the recovery was not as complete.

**Figure 15:**
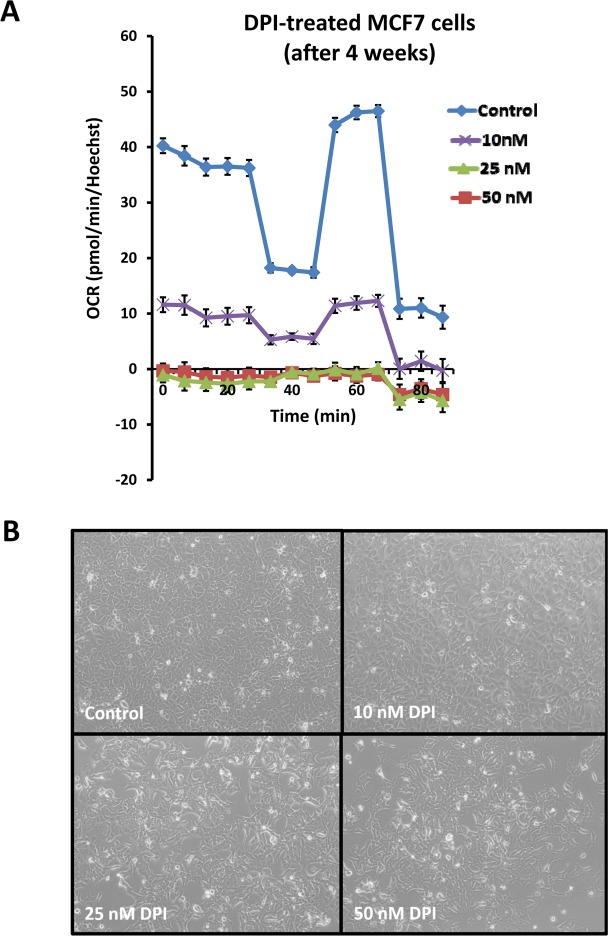
Long-term treatment with DPI is surprisingly “non-toxic” To determine the long-term effects of DPI (10, 25 and 50 nM), MCF7 cells were cultured for an entire month, in the presence of the drug. Then, their mitochondrial respiration was assessed by metabolic flux analysis. Panel (**A**) shows that all three drug concentrations show near complete inhibition of mitochondrial respiration. Panel (**B**) illustrates the morphology of cells after 4 weeks of DPI treatment. Note that the morphology and density of the cells is relatively unchanged, especially at a DPI concentration of 10 nM. Media with DPI or vehicle alone was replaced every 2 to 3 days, during the period of 1 month. Cells undergoing long-term treatment with DPI were also successfully passaged, after harvesting by standard trypsinization techniques.

Surprisingly, long-term treatment with DPI was also relatively “non-toxic”. To determine the long-term effects of DPI (10, 25 and 50 nM), MCF7 cells were cultured for 1 month, in the presence of the drug. Then, mitochondrial respiration was assessed. Figure 15A illustrates that these concentrations all show near complete inhibition of respiration. Importantly, at a DPI concentration of 10 nM, the morphology and density of the cells remains unchanged (Figure 15B).

## DISCUSSION

### DPI targets mitochondrial metabolism and eliminates CSCs

In this report, we conducted a high-throughput drug screen to discover novel mitochondrial inhibitors. More specifically, this screening assay was engineered to select chemical entities that potently reduce ATP levels, but do not induce cell death. With this strategy, DPI was identified a top hit compound. More specifically, DPI induced a state of metabolic-quiescence, which potently prevented CSC propagation (IC-50 = 3.2 nM). Complementary findings were also provided using well-established CSC markers (CD44 and CD24). Surprisingly, DPI treatment selectively depleted the CSC sub-population (CD44+/CD24-) from the total cancer cell population. We also quantitatively evaluated how DPI changes the metabolic phenotype of cells, by using the Seahorse XFe96 metabolic flux analyzer. For example, DPI, at a concentration of only 10 nM, effectively inhibited OXPHOS and ultimately ATP production, by more than 90% overall. As a consequence, DPI treatment induced a reactive glycolytic phenotype, resulting in the production of high levels of L-lactate. The metabolic effects of DPI had a rapid onset and could be detected even after only 1 hour. In addition, the effects of DPI appeared to be largely reversible, probably because low-dose DPI failed to induce mitochondrial ROS production. Basal respiration was restored to nearly 100% after 24 hours of treatment, followed by 24 hours of recovery, at a concentration of 10 nM. To assess the potential long-term effects of DPI, cells were cultured for up to 1 month with DPI treatment. During long-tern culture, little or no mitochondrial activity was observed; however, the cells remained viable.

In summary, DPI represents a new class of mitochondrial inhibitors that induced a metabolically quiescent state, which progressively depleted both i) ATP levels and ii) the CSC sub-population. As a result, our current findings have important therapeutic implications, for targeting CSCs, while reducing potentially toxic complications. Our findings provide further evidence that it is possible to identify potent mitochondrial inhibitors to more effectively target CSCs.

### What are the precise mechanism(s) by which DPI targets CSCs?

The exact mechanism(s) by which DPI targets CSCs remains largely unknown and will require further experimental investigations.

However, DPI is thought to act by binding to and targeting flavin-containing oxidase enzymes, many of which reside within mitochondria [16–18]. There are 90 flavo-proteins that are encoded within the human genome [17,18]. It has been proposed that DPI can chemically react with FMN (flavin mononucleotide), essentially inactivating these enzymatic co-factors [18].

**Figure 16:**
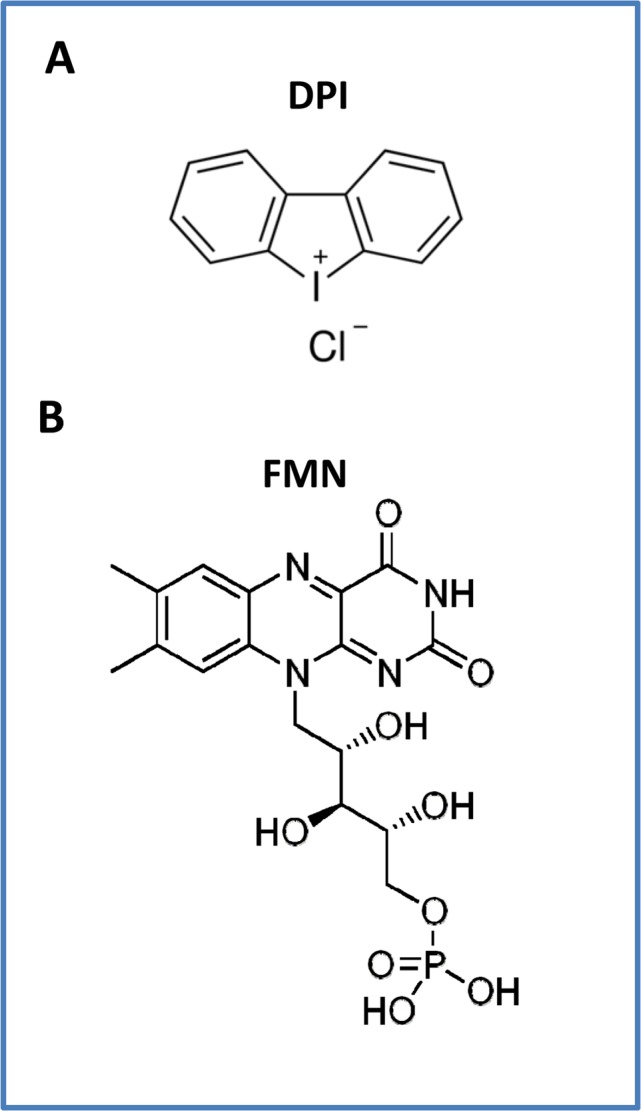
The chemical structures of (A) DPI and (B) FMN are compared It has been proposed that the effects of DPI are mediated through the general inhibition of flavo-enzymes, such as mitochondrial Complex I (NADH dehydro-genase), via the targeting of FMN. The three known flavin-containing protein components of Complex I are: NDUFV1 (51 kD), NDUFV2 (24 kD) and NDUFV3 (10 kD). It has been suggested that DPI chemically reacts with FMN, interrupting its function and impairing electron transport. In the human genome, there are ∼90 flavo-proteins; more than two-thirds require FAD, while only ∼15% require FMN. Flavo-proteins are very often localized to the mitochondria, because of their role in redox reactions. Nearly all flavo-proteins (∼90%) catalyze some form of redox reaction.

A comparison of the structures of DPI and FMN is shown in Figure 16.

Importantly, flavin-containing enzymes include three protein components of mitochondrial Complex I, which are NDUFV1 (51 kD), NDUFV2 (24 kD) and NDUFV3 (10 kD) [16,17]. However, SDHA is also a flavo-protein that is part of both mitochondrial Complex II and the Krebs cycle. As such, DPI has been proposed to act by inhibiting the mitochondria, mainly at the levels of Complex I and II [18].

Using GeneCards as a bioinformatic reference tool, we estimate that ∼70% of all flavin-containing gene products are localized to mitochondria. This may explain the exceptional potency of DPI, in targeting mitochondrial function in the low nano-molar range (IC-50 ∼ 5 to 10 nM).

**Figure 17:**
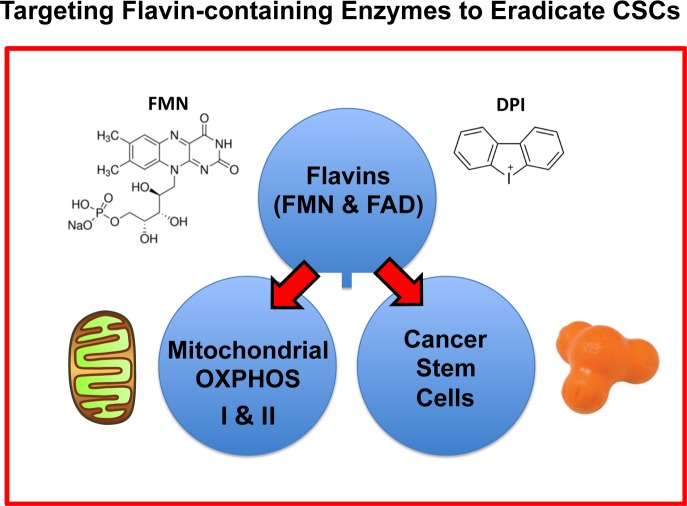
Targeting flavin-containing enzymes eradicates CSCs Flavins (FMN, FAD and Riboflavin) have been independently used as markers for high mitochondrial OXPHOS or increased CSC activity. However, it remained unknown whether flavins were selectively required for CSC propagation. Here, we showed that DPI, which is known to specifically target flavin-containing enzymes, behaves as a powerful mitochondrial OXPHOS inhibitor and successfully eradicates CSCs with high potency, in the low nano-molar range. Therefore, these findings provide the first proof-of-concept that inhibiting flavin-containing enzymes is a new viable strategy for effectively targeting CSCs.

Therefore, we speculate that the actions of DPI may be via the induction of a mitochondrial deficiency in FMN or FAD or by inactivating flavin-containing enzymes in CSCs (Figure 17). In this context, it is interesting to note that Riboflavin (Vitamin B2) is the biochemical precursor of FAD and FMN. Moreover, Riboflavin is auto-fluorescent and has been shown to correlate with both i) high mitochondrial OXPHOS capacity [19] and ii) increased CSC activity [20]. This property should also allow us to monitor the response to DPI therapy, by using the auto-fluorescent (AF) imaging of Flavins (Riboflavin, FMN and/or FAD), to visualize CSCs and cellular heterogeneity within the tumor tissue [21].

An additional mechanism for DPI's effects on mitochondria has also been proposed. In this scenario, DPI inhibits ROS production (i.e., superoxide anion), by preventing reverse electron transport from succinate at mitochondrial Complex I [22], without affecting forward electron transport. This essentially means that DPI is preventing the production on an unwanted side reaction, which contributes to unnecessary ROS production and cellular damage, during mitochondrial respiration.

### A Riboflavin-deficiency targets mitochondrial complexes I & II, inhibiting OXPHOS

Since DPI targets flavin-containing enzymes, its effects on mitochondrial function may be explained by the pharmacological induction of an acute Riboflavin (Vitamin B2) deficiency. Consistent with this hypothesis, when mammalian HepG2 cells were cultured in Riboflavin-free media, proteomics analysis revealed that key components of mitochondrial complex I (NDUFS1; NDUFV2) and complex II (SDHA) were significantly reduced (by up to 5-fold), as were many other mitochondrial-related proteins, such as AIFM1, DLD, MCAD and NQO1 [23]. Thus, a Riboflavin-deficiency directly targets the steady-state expression levels of OXPHOS-related proteins of mitochondrial complex I and II, as predicted. However, it should be noted that Riboflavin also plays a wider role in mitochondrial metabolism, by promoting fatty acid oxidation (FAO), as well as functionally stimulating mitochondrial biogenesis and driving the proper folding of complex IV [24–29].

Furthermore, dietary restriction of Riboflavin significantly reduced tumor burden (both lesion number and size), in a spontaneous model of murine mammary tumorigenesis (C3H-mice) [30,31]. Similarly, DPI treatment significantly reduced tumor growth in mice xenografted with colon cancer cell lines [32]. However, these investigators did not evaluate the effects of Riboflavin or DPI, on the status of the CSC population.

### Possible implications for aging

Mitochondria have been directly implicated in the process of aging. However, their exact role remains a hotly-debated topic. Nevertheless, it is interesting to speculate that DPI could be used to keep normal cells in a state of metabolic-quiescence or “suspended animation”, akin to hibernation, which might be extremely useful in slowing or reversing the aging process.

In support of this assertion, previous studies in *C. elegans* have shown that DPI prevents the accumulation of lipofuscin (an aging-associated by-product or marker), during the response to oxidative stress [33]. This intriguing possible use for DPI should be explored further.

### Targeting other vitamins for anti-cancer therapy: Anti-folates are a successful therapeutic strategy for targeting rapidly-dividing cells and infectious parasites

Is there any evidence that targeting the metabolism of other vitamins can be used as a successful anti-cancer strategy? The best example is Vitamin B9, also known folic acid or folate.

Anti-folates are anti-metabolites that block or disrupt the actions of folate. Most anti-folate drugs exert their effects by targeting dihydrofolate reductase (DHFR). Folate serves as a co-factor for many biosynthetic enzymes (i.e., methyltransferases) that drive methionine, serine, purine and thymidine biosynthesis. Examples of anti-folate drugs that are FDA-approved include: Methotrexate; Pemetrexed; Proguanil; Pyrimethamine; and Trimethoprim.

The actions of anti-folates preferentially target rapidly dividing cells, especially during DNA-synthesis (the S-phase of the cell cycle). Currently, both Methotrexate and Pemetrexed are routinely used for the treatment of various cancer types, such as osteosarcoma, non-small cell lung carcinoma, mesothelioma and hematologic malignancies. Therefore, anti-folate therapy is considered as a successful strategy for treating cancer and various infectious parasitic diseases, such as malaria, toxoplasmosis and pneumocystis pneumonia.

However, anti-folates also have significant side effects, because they also affect the proliferation of normal cells, leading to nausea, vomiting, abdominal pain, agranulocytosis and aplastic anemia (bone marrow suppression).

## CONCLUSIONS

In summary, we have identified DPI as a mitochondrial inhibitor for the specific targeting of CSCs, in a heterogeneous population of cells.

DPI is one of the most potent and highly-selective CSC inhibitors discovered to date. For example, DPI is ∼30 times more potent than Palbociclib (IC-50 ∼ 100 nM), which is already FDA-approved [34].

**Figure 18:**
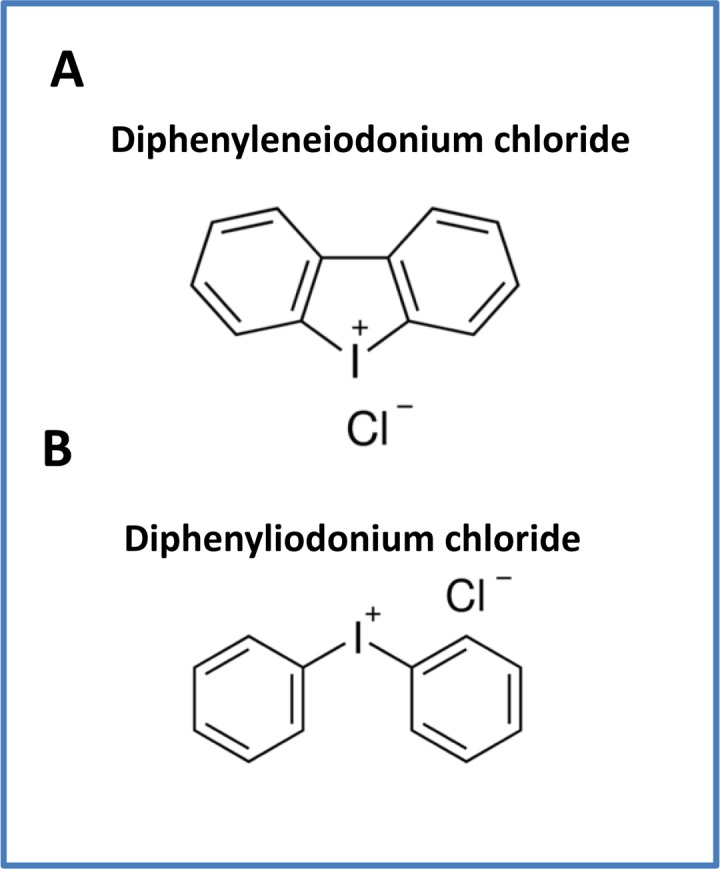
Comparison of the structures of (A) Diphenyleneiodonium (DPI), with the related compound (B) Diphenyliodonium chloride Note the key similarities between these two chemical structures. Both of these classes of molecules target flavin-containing proteins.

This provides a new impetus to create novel analogues of i) DPI (Diphenyleneiodonium chloride) and ii) DPI-related compounds (Diphenyliodonium chloride) (Figure 18), using medicinal chemistry, to optimize this very promising and potent anti-CSC activity. We propose to call these new molecules “Mitoflavoscins”.

In the broadest sense, “Mitoflavoscins” would be considered as any small molecule(s) or peptide(s) that bind to flavin-containing enzymes [or actively depletes FMN, FAD or Riboflavin from cells] and, as a consequence, inhibits mitochondrial function.

## MATERIALS AND METHODS

### Materials

MCF7 cells were purchased from ATCC (American Type Culture Collection). Gibco-brand cell culture media (DMEM) was purchased from Life Technologies

### Phenotypic drug screening, with a metabolic ATP-depletion assay

MCF7 cells (6,000 cells/well) were plated into black clear-bottom 96-well plates and incubated overnight before treatment. Then, a sub-set of the Tocriscreen compound library (560 compounds) were subjected to phenotypic drug screening at a concentration of 20 μM, to identify which compounds functionally induce ATP-depletion, before inducing cell death. Subsequently, positive hits were re-screened at 10 μM, to identify the top 11 compounds that most potently induced ATP-depletion. Compounds were tested after 72 hours of incubation and experiments were performed in triplicate. After treatment, media was aspirated from the wells and plates were washed with warm PBS (supplemented w/ Ca^2+^ and Mg^2+^). Then, cells were incubated with a Hoechst 33342 (Sigma) staining solution (10 μg/ml) for 30 min and washed with PBS. Fluorescence was read with a plate reader using excitation/emission wavelengths at 355/460-nm. Then, the CellTiter-Glo luminescent assay (Promega) was performed to measure metabolic activity (ATP content) in the very same wells that were treated with a given compound. Assays were performed according to the manufacturer's protocol. Fluorescence (Hoechst staining) and luminescence intensities (ATP content) were normalized to vehicle-alone treated controls and were displayed as percentages.

### Cell viability assay

The Sulphorhodamine (SRB) assay is based on the measurement of cellular protein content. After treatment for 72 hours with DPI in 96-well plates, the cells were fixed with 10% trichloroacetic acid (TCA) for 1 hour in the cold room, and were dried overnight at room temperature. Then, cells were incubated with SRB for 15 min, washed twice with 1% acetic acid, and air dried for at least 1 hour. Finally, the protein-bound dye was dissolved in a 10 mM Tris, pH 8.8, solution and analyzed using a plate reader at 540-nm.

### Metabolic flux analysis using the seahorse XFe96

Extracellular acidification rates (ECAR) and real-time oxygen consumption rates (OCR) for MCF7 cells were determined using the Seahorse Extracellular Flux (XF96) analyzer (Seahorse Bioscience, MA, USA). MCF7 cells were maintained in DMEM supplemented with 10% FBS (fetal bovine serum), 2 mM GlutaMAX, and 1% Pen-Strep. 8,000 cells per well were seeded into XF96-well cell culture plates, and incubated overnight at 37°C in a 5% CO_2_ humidified atmosphere. Next day, cells were washed in pre-warmed XF assay media (for OCR measurement, XF assay media was supplemented with 10mM glucose, 1mM Pyruvate and adjusted at pH 7.4). Cells were then maintained in 175 μL/well of XF assay media at 37°C, in a non-CO2 incubator for 1 hour. During incubation, 25 μL of of 80mM glucose, 9μM oligomycin, 1M 2-deoxyglucose (for ECAR measurement) and 25 μL of 10μM oligomycin, 9μM FCCP, 10μM rotenone, 10μM antimycin A (for OCR measurement) in XF assay media was loaded into the injection ports of the XFe-96 sensor cartridge. During the experiment, the instrument injected these inhibitors into the wells at a given time point, while ECAR/OCR was measured continuously. ECAR and OCR measurements were normalized by protein content (Sulphorhodamine B assay). Data sets were analyzed by XFe-96 software, using one-way ANOVA and Student's t-test calculations. All experiments were performed in triplicate.

### Measurement of L-lactate levels in cell culture media

Culture media were collected, centrifuged and analyzed with ISCUS-flex Microdialysis Analyzer after treatment of MCF7 cells with various concentrations of DPI for 1, 3 or 5 days. First, calibration of the instrument was performed by samples provided by the manufacturer. Then L-lactate levels were measured and normalized to samples taken from MCF7 cells treated with vehicle only.

### Mammosphere formation assays

A single cell suspension of MCF7 cells was prepared using enzymatic (1x Trypsin-EDTA, Sigma Aldrich) and manual disaggregation (25 gauge needle. Cells were then plated at a density of 500 cells/cm^2^ in mammosphere medium (DMEM-F12/ B27 / 20-ng/ml EGF/PenStrep) in non-adherent conditions, in culture dishes coated with (2-hydroxyethylmethacrylate) (poly-HEMA, Sigma). Cells were grown for 5 days and maintained in a humidified incubator at 37°C at an atmospheric pressure in 5% (v/v) carbon dioxide/air. After 5 days in culture, spheres >50 μm were counted using an eye-piece graticule, and the percentage of cells plated which formed spheres was calculated and is referred to as percent mammosphere formation, normalized to vehicle-alone treated controls. Mammosphere assays were performed in triplicate and repeated three times independently.

### Mitochondrial ROS measurement

Production of superoxide by mitochondria was measured by the MitoSOX Red mitochondrial super-oxide indicator (ThermoFisher Sci., M36008). 3×10^5^ MCF7 cells/well were plated in 6-well plates in complete media supplemented with 10% heat-inactivated FBS. The next day, cells were treated with DPI (5, 50 nM) or Rotenone (0.5 μM) for 24 hours. Vehicle alone (DMSO) for control cells were processed in parallel. At least 30,000 events were recorded by FACS using Fortessa (BD Bioscience). Importantly, three biological replicates (repeats) were analyzed in independent experiments. Results are the average of the mean of each experiment and are expressed as percentages of mean fluorescence intensity normalized to control. `Comp` in the title of X-axis means data were normalized with autocalculated compensation. ^*^ p<0.05, ^**^ p<0.01, ns not significant. One-way ANOVA was used with Dunnett's multiple comparisons test. A Brown-Forsythe test was used to test the equality of group variances.

### CD44/CD24 analysis

1 × 10^5^ MCF7 cells were plated in 6-well plates in complete media supplemented with 10% heat-inactivated FBS. Next day, cells were treated with DPI (5, 10, 50 nM) for 5 days. Vehicle alone (DMSO) control cells were processed in parallel. Briefly, 30,000-50,000 live cells, as identified by 7-AAD dye staining, were analyzed for CD24/CD44 expression. We employed CD24 (IOTest CD24-PE, Beckman Coulter) and CD44 (APC mouse Anti-Human CD44, BD Pharmingen) antibodies for FACS-analysis, using the BD LSR Fortessa (BD Bioscience). Results are the average of three biological replicates (repeats) and are expressed as percentages of mean fluorescence intensity, normalized to the control. ^*^ p<0.05, ^**^ p<0.01, ^***^ p<0.001. One-way ANOVA was used with Bonferroni's multiple comparisons test.

### Apoptosis analysis

Cell death was quantified by flow cytometry using propidium iodide (PI) and Annexin V-FITC, essentially as previously described [35]. Briefly, 300,000 MCF7 cells were plated in 6 multiwell plate in complete media supplemented with 10% HiFBS. Next day, cells were treated with DPI (5, 10, 50 nM). Vehicle alone (DMSO) for control cells were processed in parallel. After 24 hours, cells were harvested and washed in cold phosphate-buffered saline (PBS). Cells were recentrifuged and supernatants were discarded. Then, cells were re-suspended in 100 μl of annexin-binding buffer. Then, the annexin–FITC conjugate (5 μl) and PI (1 μL) were added and incubated in the dark at room temperature for 15 min. After the incubation period, reaction was stopped by adding 400 μL of annexin-binding buffer. Cells were then analyzed by flow cytometry using a PE Texas Red signal detector for PI staining and a FITC signal detector to detect Annexin V binding. 30,000 events were recorded by FACS using LSRII. Results are the average of three biological replicates analyzed and expressed as mean fluorescence intensity.

### Statistical analyses

Statistical significance was determined using the Student's t-test (unless stated otherwise); values of less than 0.05 were considered significant. Data are shown as the mean ± SEM.
